# From Metabolism to Genetics and Vice Versa: The Rising Role of Oncometabolites in Cancer Development and Therapy

**DOI:** 10.3390/ijms22115574

**Published:** 2021-05-25

**Authors:** Emanuela Di Gregorio, Gianmaria Miolo, Asia Saorin, Agostino Steffan, Giuseppe Corona

**Affiliations:** 1 Immunopathology and Cancer Biomarkers Unit, Centro di Riferimento Oncologico di Aviano (CRO), IRCCS, 33081 Aviano, Italy; emanuela.digregorio@cro.it (E.D.G.); asaorin@cro.it (A.S.); asteffan@cro.it (A.S.); 2 Medical Oncology and Cancer Prevention Unit, Centro di Riferimento Oncologico di Aviano (CRO), IRCCS, 33081 Aviano, Italy; gmiolo@cro.it

**Keywords:** oncometabolites, cancer, metabolism, epigenetics, therapy, metabolomics

## Abstract

Over the last decades, the study of cancer metabolism has returned to the forefront of cancer research and challenged the role of genetics in the understanding of cancer development. One of the major impulses of this new trend came from the discovery of oncometabolites, metabolic intermediates whose abnormal cellular accumulation triggers oncogenic signalling and tumorigenesis. These findings have led to reconsideration and support for the long-forgotten hypothesis of Warburg of altered metabolism as oncogenic driver of cancer and started a novel paradigm whereby mitochondrial metabolites play a pivotal role in malignant transformation. In this review, we describe the evolution of the cancer metabolism research from a historical perspective up to the oncometabolites discovery that spawned the new vision of cancer as a metabolic disease. The oncometabolites’ mechanisms of cellular transformation and their contribution to the development of new targeted cancer therapies together with their drawbacks are further reviewed and discussed.

## 1. The Rebirth of Cancer Metabolism 

In early 1920, Otto Warburg made a very significant observation still acknowledged 98 years later that remarkably contributed to the recent renaissance of cancer metabolism research. During biochemical studies on sea urchin eggs, he noted a significant increase in oxygen consumption during fertilization [1], demonstrating for the first time that cellular replication and growth can induce changes in metabolism [2,3]. In further investigations on mice hepatoma tissue slices supplemented with glucose, he observed a 70-fold increase of lactic acid production independently from the presence of oxygen that would be named aerobic glycolysis or the “Warburg effect” [4]. Warburg attributed the cause of the impairment of cellular respiration to dysfunctional mitochondria and became convinced that defective metabolism was the main driver of carcinogenesis [2] as declared to the German Central Committee for Cancer Control in Stuttgart 1955 “… there is today no other explanation for the origin of cancer cells, either special or general. From this point of view, mutation and carcinogenic agent are not alternatives, but empty words, unless metabolically specified…” [5]. From its early beginning, Warburg’s theory found many opponents that considered the “Warburg effect” only an adaptation to hypoxic conditions due to the poor vascularization of the tumours. The biochemist Weinhouse confuted Warburg’s theory showing that cancer cells were able to accomplish oxidative phosphorylation like normal cells and, unlike Warburg, he sustained that dysfunctional mitochondria were not the cause but the consequence of upregulated glycolysis [6]. However, more quickly than expected, the debate ended and Warburg’s idea was considered old-fashioned. Meanwhile the genetic model of cancer based on the Somatic Mutation Theory (SMT) originally proposed by Theodor Boveri [7] became the most attractive. The attention on cancer metabolism was definitively taken away by the discoveries of oncogenes and tumour suppressor genes starting from the *SRC* oncogene initially isolated in Rous sarcoma retrovirus [8,9,10,11] and later in human cells [12]. The *SRC* oncogene was followed by *MYC, ERBB/EGFR*, and *RAS* oncogenes [13,14,15,16,17] whose identification in diverse human cancers [18,19] consolidated the view of cancer as a genetic disease. Only at the end of the 90s was an unexpected link found between the genes involved in cell proliferation and cellular energy metabolism that led to reconsideration of the importance of metabolism and Warburg’s theory in cancer development. In 1997 Dang et al. demonstrated that the transcription factor MYC, well known for its role in cell cycle and apoptosis, directly affected the expression of the lactate dehydrogenase-A gene (*LDH-A*), turning on the Warburg effect [20]. Independently, Craig Thompson et al. identified the cause that sustained the aerobic glycolysis in the alteration of the protein kinase B (Akt) signalling pathway commonly activated in human cancers in response to different transcription factors [21] including p53, hypoxia-inducible factor (HIF) and nuclear factor-κB (NF-κB), which were further found to be involved in cancer metabolic reprogramming [22,23,24]. The breakthrough for the Warburg revival coincided with the finding of specific mutated genes encoding for enzymes of the tricarboxylic acid (TCA) cycle as the succinate dehydrogenase (SDH) [25,26] and fumarate hydratase (FH) [27]. The loss of function (LoF) of these mitochondrial enzymes lead to the accumulation of their respective substrates, as succinate and fumarate, in different human cancer types, supporting the original role of mitochondria in cancer development. The observation that two mitochondrial enzymes could act as classic tumour suppressors [25,28] instilled the idea that oncogenes and tumour suppressor genes can also express their functions exclusively by reprogramming cellular metabolism. A few years later, the discovery of a specific defect in the enzymatic activity of the mitochondrial isocitrate dehydrogenase (IDH) [29,30,31] led to the tumour accumulation of 2-hydroxyglutarate (2-HG), further proving the importance of specific metabolites in tumorigenesis starting the oncometabolites era. 

## 2. Oncometabolites: The Emerging of a New Paradigm

The cellular accumulation of succinate, fumarate and 2-HG promotes and sustains specific metabolic phenotypes that induce cancer development and growth. For these characteristics, they take the name of oncometabolites, defined as small molecules whose abnormal cellular accumulation is able to activate oncogenic signalling and promote a milieu favourable for the tumorigenesis. Interestingly, all of them are the result of genetic mutations encoding enzymes of the TCA cycle supporting the essential role of mitochondria as signalling hubs for key biological functions in both normal and cancer cells fate [32]. Despite oncometabolites cellular accumulation is associated with gene mutations, their oncogenic mechanisms go beyond genetics since they can directly act at all omics levels, altering cell signalling and regulating gene expression and protein functions as unique cancer phenotype modulating agents. 

### 2.1. The Succinate

**Figure 1:**
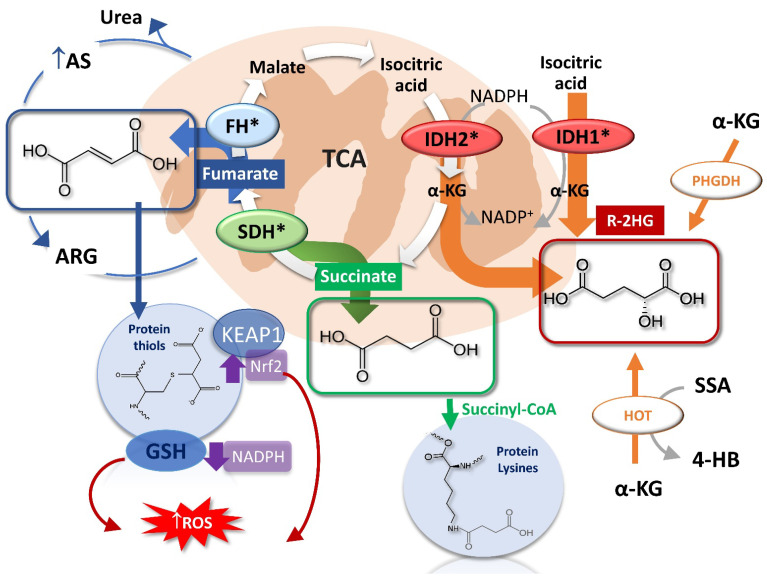
Oncometabolites production and reactions. Loss of function of mutated SDH and FH enzymes leads to the accumulation of succinate and fumarate that triggers post-translational proteins modifications such as succinylation and succination, respectively. The R-2HG derives mainly from the neomorphic catalytic activity of the mutated mitochondrial IDH2 and cytosolic IDH1, and less from non-canonical reactions involving the PHDGH and HOT enzymes. FH, fumarate hydratase; SDH, succinate dehydrogenase; α-KG, α-ketoglutarate; R-2HG, R-2-hydroxyglutarate; PHDGH, phosphoglycerate dehydrogenase; HOT, hydroxyacid-oxoacid-transhydrogenase; 4-BH, 4- hydroxybutyrate; SSA, succinic semialdehyde; AS, argininosuccinate; ARG, arginine.

The identification of germline *SDHD* gene mutations both in hereditary paragangliomas (PGLs) [25] and pheochromocytomas (PCCs) [26] moved attention on the role of mitochondrial metabolism in cancer development again. The SDH genes (*SDHA*, *SDHB*, *SDHC*, *SDHD*) encode the subunits of the succinate dehydrogenase tetrameric complex also known as mitochondrial complex II, which is involved in the TCA cycle as well as in the aerobic electron transport chain (ETC) [33] (Figure 1). This latter is constituted by four subunits (SDHA, SDHB, SDHC, SDHD) assembled in two protein complexes. The SDHA and SDHB constitute the catalytic subunits responsible for the oxidative conversion of succinate to fumarate, while the SDHC and SDHD subunits have only a structural role as membrane anchor [34]. The defects in SDH activity raised from germline mutations on chromosome 1 or 11 can include missense, nonsense, frameshift, splicing defect, and deletion/insertion [35,36]. The LoF of SDH occurs by the loss of heterozygosity (LOH) due to the second allele deletion that arrests the succinate- fumarate conversion [37]. Cellular metabolomics investigations pointed out the specific accumulation of the succinate [38,39,40,41,42] in tumours that carry the LOH for the *SDH*x mutated genes including renal cell carcinoma, gastrointestinal stromal tumour, pituitary adenomas pancreatic neuroendocrine tumours [43,44,45,46,47,48] and PGLs [49]. The SDH immunohistochemistry (IHC) staining using specific SDHB antibodies has been proposed as test for the screening of *SDH*x mutations since the loss of SDHB subunit is correlated with any *SDHx* mutations [50]. However, the IHC presents some drawbacks linked to the heterogenic or weak diffuse SDHB immunostaining that may increase the risk for false-negative or positive cases [49,51,52]. Therefore, succinate accumulation or the phenotypic succinate/fumarate ratio, measured by liquid chromatography-mass spectrometry, has been proposed to improve the sensitivity and specificity of *SDH*x detection [41]. The LOH for the *SDH*x can be reviled non-invasively in vivo by proton ^1^H-MRS tomography through measuring differential succinate accumulation [53,54].

The common biochemical consequence of the succinate accumulation was the increased production of ROS likely due to the role played by the SDH in the ETC [55,56]. Different evidences showed an increase of oxidative stress in SDH mutant tumours which was associated with genomic instability and tumorigenesis [55,57,58,59]. However, succinate can also leave mitochondria and exert different effects on both the cytosolic and nuclear key enzymes directly involved in malignant transformation [60] as described in the following section. 

### 2.2. The Fumarate

The *FH* gene encodes a key TCA cycle enzyme and its germinal mutations at chromosome locus 1q43 have been associated with the decrease of enzyme activity and fumarate cellular accumulation [61]. Among the *FH* mutations, the missense and frameshift [62,63] are the most common found in uterine fibroids, hereditary leiomyomatosis, and renal cell carcinoma syndrome (HLRCC) [27] and also in PGLs and PCCs [64,65]. These mutations indeed lead to significant reduced FH activity [66] or to the premature truncation of the protein [67]. The missense mutations mainly involved the conserved enzyme’s active site or subunits important for inter-intra interactions and protein stability [66,68]. The FH is a homotetrameric enzyme localized in both mitochondria and cytosol where it is involved in the reversible hydration of fumarate to malate as well as in the catabolism pathways of amino acids [69] (Figure 1). The early diagnosis of tumour *FH* genetic defects could be clinically detected by IHC of the protein or by metabolomics investigations to search for specific fumarate accumulation [41,70]. The FH immunostaining integrated with the IHC for succinated proteins [71,72,73,74] is the most used diagnostic test for detecting mitochondrial FH dysfunction identified by FH negative and 2-succinocysteine positive staining [72,74,75]. The FH and succinated proteins IHC are generally classified with “0” score for negative staining (total loss), “1+” or “2+” score for focal or diffuse staining with weak or strong intensity, respectively (partial loss) [74]. The fumarate/malate ratio could be also used for diagnostic purposes as well as other specific metabolic features consequent to fumarate cellular accumulation, including the reversal induction of the argininosuccinate lyase (ASL) activity [76,77].

The FH LoF induces a significant fumarate accumulation that leads to post-translational modifications, affecting proteins functions out of mitochondria and causing chromatin modulations altering epigenetic status and gene expression that drives malignant transformation through specific biochemical mechanisms detailed in further sections.

### 2.3. The R-2-Hydroxyglutarate 

The involvement of R-2HG metabolite in cancer was ignored until 2008 when Parsons et al. sequenced over 20,000 genes in glioblastoma [31] finding in 12% of patients a somatic mutation in the isocitrate dehydrogenase (*IDH*) gene. Different metabolomics analyses in tumours tissues, as well as in cerebrospinal fluid, blood, and urine, demonstrated that the *IDH* gene mutation is associated with a huge cellular accumulation of the R-2HG [78,79,80,81,82,83,84]. Further studies found the same mutation also in II-III grade gliomas, in secondary glioblastoma [29,30], as well as in extra-brain cancers such as human acute myeloid leukaemia (AML) [85], intrahepatic cholangiocarcinoma [86], chondrosarcomas [87] and breast carcinoma [88,89,90]. The IDH is an important enzyme involved in the TCA cycle responsible for the reversible oxidative decarboxylation of isocitrate to α-ketoglutarate (α-KG) (Figure 1). The IDH enzyme is present in three distinct isoforms that differ for localization and co-factors dependence: the homodimers IDH1 and IDH2 use nicotinamide adenine dinucleotide phosphate (NADP) cofactor and are localized in cytosol and mitochondria, respectively, while the heterotrimeric IDH3 isoform is dependent on NAD cofactor which confers a regulatory activity in function of the cell energy status and catalyses the forward reaction from isocitrate to α-KG. The most common cancer mutations involve the IDH1 and IDH2 isoforms and resulted to be mutually exclusive [91]. Their prevalence is different according to the cancer type since it was found that *IDH1* has higher incidence rate than *IDH2* in brain cancers [91,92], while in AML they are equally common [93], likely reflecting the differential tumour metabolic needs.

The *IDH* mutations concern the substitution of the arginine R132 (for IDH1), and R172 or R140 (for IDH2) with a histidine residue. The arginine substitution decreases the affinity for isocitrate substrate, resulting in a significantly slower rate of conversion to α-KG. Also, it increases the affinity for NADPH that confers a neomorphic activity to the enzyme allowing the further stereospecific conversion of α-KG into the R-2HG enantiomer [79]. The concentration of R-2HG metabolite in glioma cells carrying the mutated IDH1/2 can reach ~30 millimolar [79,94,95] saturating the mitochondrial R-2HG dehydrogenase responsible for its cellular removal [96]. Interestingly, even the IDH wild type tumour produced the enantiomer R-2HG, however its concentration resulted 100 fold less than *IDH* mutated tumours [96]. The IDH heterozygous mutation was found to be the necessary condition to reach such high R-2HG levels [97]. The *IDH* mutations have remarkable clinical utility allowing a better gliomas classification [98] that improves the current diagnosis and prognosis of this cancer disease [99,100,101,102]. Numerous investigations have focused on R-2HG as a surrogate biomarker for identifying *IDH* mutations. In AML the measurement of R-2HG can be carried out directly in patients’ serum or plasma where it resulted notably correlated with *IDH* mutations [103,104]. However, despite the good diagnostic power, the serum R-2HG level in AML has poor prognostic relevance not having been found associated with the overall clinical outcomes [105,106]. In glioma, advances in magnetic resonance spectroscopy (MRS) allowed researchers to effectively measure 2HG in vivo with good correlation with *IDH* mutation status [107,108,109,110,111,112], whereas MRS can measure total 2HG at the tissue level, chromatography coupled with mass spectrometry enables the specific quantification of R- and S-stereoisomers in the cerebrospinal fluid, serum and urine. However, its application has produced no concordant results since some studies found a positive correlation between high 2HG levels and mutation status [81,82,83,113,114] while others reported no difference between wild-type and mutant patients [115,116]. These discrepancies could be associated with the lack of standardized analytical methods or depend on patients’ pathological status since the blood-brain barrier integrity may be differentially compromised in gliomas patients [117,118], affecting the amount of 2HG that reaches blood and urine. 

Beyond the *IDH*-mutated production, the cellular R-2HG was found to have other sources that further highlight its active role in cancer development [119] (Figure 1). In breast cancer, the oncogene phosphoglycerate dehydrogenase (PHGDH) was reportedly able to accumulate both S/R-2HG enantiomers from α-KG [120] as well as the overexpression of the mitochondrial hydroxyacid-oxoacid-transhydrogenase (HOT), which produces R-2HG as a consequence of the 4- hydroxybutyrate to succinic semialdehyde conversion [121,122] (Figure 1). Moreover, R-2HG seems to be derived from specific immune cells such as activated T helper (Th) 17 when they metabolically switch from oxidative phosphorylation to aerobic glycolysis in the response against the tumour [123]. All this evidence has reinforced the hypothesis that R-2HG involvement in tumorigenesis may be independent from *IDH* mutations, revealing insights on novel cancer targets. 

## 3. Oncometabolites Mechanism of Cancer Induction 

**Figure 2:**
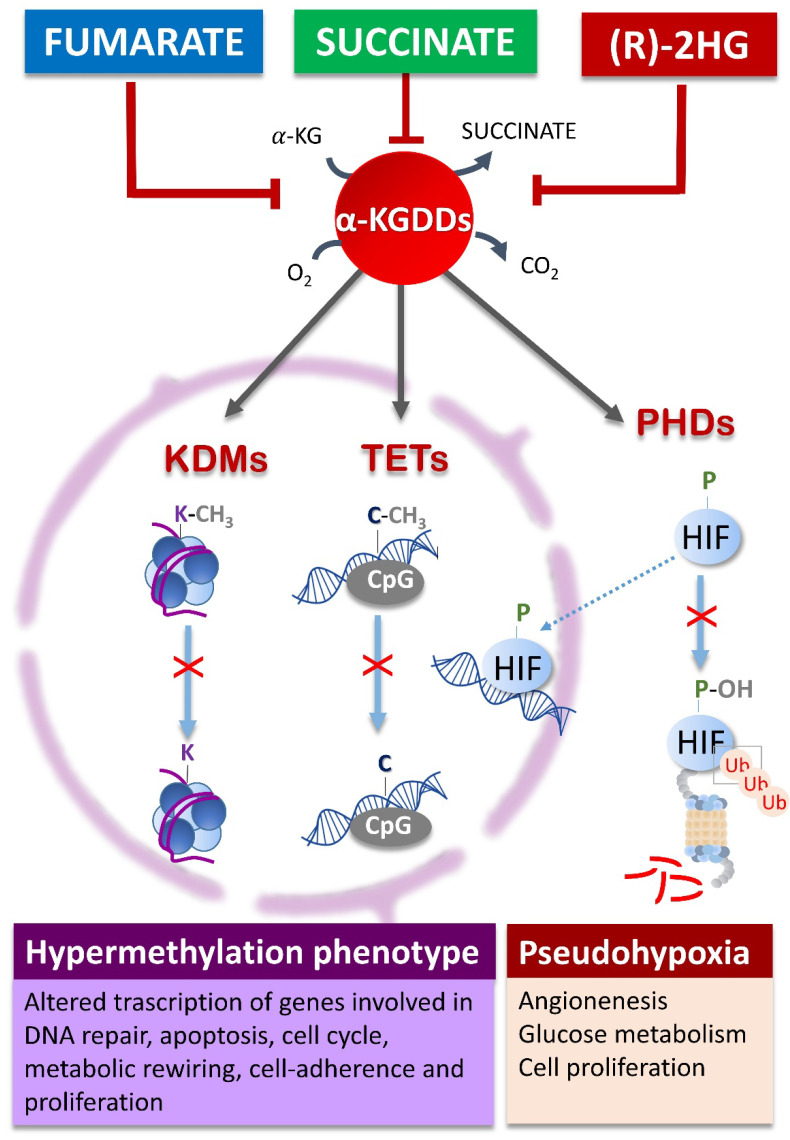
Oncometabolites epigenetic and pseudohypoxia effects. Oncometabolites act as competitive inhibitors of αKG-dependent dioxygenases (αKGDDs) such as the KDMs and TETs families responsible for the modulation of chromatin by demethylation of histones and DNA CpG islands, respectively. The inhibition of PHDs blocks the HIF proline hydroxylation for the ubiquitin-proteasome degradation leading to HIF stabilization and activation of the hypoxia signalling pathway establishing a pseudo-hypoxic phenotype. KDMs, lysine histone demethylases; TETs, ten-eleven translocation; K, lysine; C, cytosine; P, proline; PHDs, prolyl hydroxylases; HIF, hypoxia-inducible factor; Ub, ubiquitin.

The cellular accumulation of oncometabolites initiates carcinogenesis and sustains the invasive neoplastic phenotype by interfering with important cellular metabolic signalling at both genomic and proteomic levels [124,125]. They have a common biochemical mechanism of interference which consists in the inhibition of the activity of the cellular α-KG dependent dioxygenases (α-KGDDs) [126,127,128], a wide family of enzymes that includes about 60 different isoforms involved in the control of epigenetic modifications and hypoxia responses [129]. The α-KGDDs are Fe (II) enzymes that catalyse oxidative hydroxylations of target substrates both at transcriptional and post-transcriptional levels. The hydroxylation at the methyl group represents the first step for the final methyl removal from histones and DNA, occurring through sequential oxidative formylation and decarboxylation reactions with consumption of O_2_ and α-KG and CO_2_ and succinate production [129] (Figure 2). Oncometabolites are all structurally similar to the α-KG substrate and compete with its active binding site inhibiting the activity of α-KGDDs. The inhibition of two specific α-KGDDs, such as the ten-eleven translocation (TETs) and Jumonji domain-containing histone-lysine demethylases (KDMs), induced by the high cellular concentration of succinate, fumarate or R-2HG can have relevant epigenetic consequences being strictly involved in the modulation of the chromatin and genome methylation status [130,131,132] (Figure 2). The KDMs are directly involved in the demethylation of the histones at lysine residues [132], while TETs catalyse the DNA demethylation of 5-methylcytosine regulating the transcription of the genes [133]. Thus, the competitive inhibition of KDMs and TETs by oncometabolites induces a typical hypermethylation phenotype [126,130,134,135,136] that affects the transcription of genes involved in DNA repair (*MGMT*, *BRCA*, *ATM*), apoptosis (*DAPK*, *TMS1*), cell cycle (*p16INK4a*, *p15INK4b, Rb*, *p14ARF*), carcinogen-metabolism (*GSTP1*), and cell-adherence (*CDH1*, *CDH13*), enabling cancer growth and proliferation [137,138]. Other genes are regulated specifically by either KDM or TET isoforms. The specific inhibition of the KDM4B isoform by oncometabolites resulted in the hypermethylation at histone 3 lysine 9 (H3K9) with consequent alteration of the pathway of homology-dependent repair (HDR) that prevents the recruitment of DNA repair factors [139]. The KDM4B inhibition was also found associated with aberrant activation of mTOR pathway in *IDH* mutant tumour [140,141]. Moreover, the TET inhibition was found correlated with impaired myeloid differentiation in AML due to hypermethylation in genes containing DNA binding motifs for transcription factors important for leukemogenesis [142]. The HIF-prolyl 4-hydroxylases (PHDs) is another α-KGDD inhibited by oncometabolites. It is responsible for the regulation of cellular stability of the HIF, a transcription factor targeting several genes mainly involved in angiogenesis, glycolysis, and apoptosis [143,144]. In normoxic conditions, HIF resulted hydroxylated by PHDs at prolines residues allowing its cellular degradation through the ubiquitin-proteasome system (Figure 2). The high oncometabolites concentration prevents the HIF hydroxylation stabilizing it in the active form inducting a pseudohypoxia status characterized by increased angiogenesis and glucose metabolism [37,145,146,147,148]. Independently by HIF mechanism, fumarate and succinate can additionally enhance hypoxia signalling via TETs epigenetic activation [149] and through ABL1 up-regulation and mTOR pathway [150]. Moreover, succinate itself can upregulate vascular endothelial growth factor (VEGF) through the activation of succinate receptor G protein-coupled receptor-91 (GPR91) further promoting tumour angiogenesis [151,152]. Unlike succinate and fumarate, the role of R-2HG in the induction of pseudohypoxia is controversial. Some studies have reported that it could both activate [153,154,155] and inactivate the PHDs [148,156], while others sustain no correlation between R-2HG and HIF activation in gliomas [157,158] suggesting that R-2HG may not be the main regulator of the HIF signalling pathway. 

In addition to these features, each oncometabolite exerts distinct pathological functions that likely are associated with the specific cancer type where they accumulate. High cellular fumarate concentrations induce protein succination, a post-translational modification generated by the Michael addition of proteins’ cysteine thiols to fumarate with the production of S-(2-succino)-cysteine derivatives [159] (Figure 1). This irreversible protein modification can involve Kelch-like ECH-associated protein-1 (KEAP1) that normally exerts a negative control on the nuclear factor erythroid 2–related factor 2 (Nrf2) [160,161]. This latter is a regulator of cellular antioxidant systems and the KEAP1 succination triggers the Nrf2 related-genes up-regulation, making tumoral cells able to adapt to oxidative stress favouring cell proliferation and survival [162,163,164,165]. Conversely, fumarate succination of glutathione (GSH) causes a drop of the cellular NADPH levels which is associated with an enhancement of ROS production promoting cancer by cellular damage [166,167,168]. Another specific fumarate effect has been associated with urea cycle alteration since the fumarate accumulation reverses the ASL reaction with consequent arginosuccinate increase that makes FH-deficient cells auxotrophic for arginine [77]. Analogously to fumarate, succinate can induce succinylation of proteins, a characteristic post-translation modification mediated by succinyl coenzyme A whose level increased consequently the high cellular succinate accumulation in SDH-deficiency cells [169,170]. The nucleophilic addition of the ɛ-amino group of lysine to the succinyl-CoA generates succinyl-lysine proteins exchanging the positive charge on lysine which may alter protein structural conformational and protein-protein interactions [170]. Indeed, succinylation was reported to affect several biological protein targets such as nuclear histones [169,171] and mitochondrial proteins [172,173,174] that could alter the endoplasmic reticulum protein processing, increase the glucose metabolism and confer resistance to cell apoptosis to sustain cell growth [175,176] (Figure 1). The main sites of hypersuccinylation were identified in mitochondrial protein such as pyruvate dehydrogenase complex, ATP synthase, respiratory chain complexes I, III, and IV, cytochrome c oxidase but also in SHDB itself, with consequent impair in cellular respiration and increased recruit of BCL-2 to mitochondrial membrane preventing apoptosis [172,174,177].

Other oncogenic mechanisms specific of the R-2HG concern the selective inhibition of the α-KG-dependent branch chain amino acid transaminases 1 (BCAT1) and BCAT2 [178], decreasing glutamate levels and making cells auxotrophic for this amino acid as well as for glutamine necessary to continuously replenish the NADPH consumed by mutant IDH and essential to fuel the cellular anabolism that supports the tumour growth [179]. Whereas the inhibition of the ALKBH homolog, another α-KG-dependent enzyme, decreases the DNA repair activity accumulating DNA damage in cancer cells [180]. Both effects could explain the higher efficacy of glutaminase inhibitors and alkylating agents in *IDH* mutant cancer [181,182,183,184,185,186], as well as the best prognosis to temozolomide treatments in *IDH*, mutated glioma [181,186,187,188]. Conversely, R-2HG may induce drug resistance phenomena by inducing the overexpression of the homeobox protein NANOG increasing multidrug resistance protein 1 expression [189]. Another interesting R-2HG effect is represented by its immunosuppressive activity in the tumour microenvironment. The R-2HG can inhibit the chemotaxis mediated by signal transducer—activator of transcription 1 (STAT1) reducing the tumour infiltrating CD8+ cytotoxic T lymphocyte [190,191,192], and increasing the regulatory T-cell over the Th17 [193]. It also prevents T lymphocyte activation by suppressing the ATPase and reduces the nuclear factor of activated T cell (NFAT) expression [194,195] and inactivates the complement-mediated lysis and phagocytosis in gliomas cells [196]. All these aspects seem to suggest that R-2HG could contribute to tumour escape from the immune system surveillance and should be taken into account when immunotherapy strategies are considered in gliomas treatments.

## 4. Targeted Therapies

The role of oncometabolites in cancer has stimulated the development of novel targeted therapies that are mainly addressed to the inhibition of their accumulation and/or the constraint of their metabolic and epigenetic downstream effects. The first pharmacological strategy brought to the development of specific IDH inhibitors to decrease R-2HG production. This approach has been exclusively exploited for IDH over the SDH and FH targets because the IDH mutant enzyme gains a neomorphic function different from that of wild type. Moreover, compared with succinate and fumarate, which are involved in several physiological metabolic pathways, the R-2HG apparently does not have any known relevant physiological activities [197]. These features addressed the development of numerous drugs for the mutated IDH form, which mainly acts as allosteric inhibitors (Table 1) [198,199,200,201,202,203]. Most of these drugs were designed and developed by the emerging Agios Pharmaceutical and two of them, enasidenib and ivosidenib, have found a place in clinics. 

Enasidenib (IDHIFA^®^, AG-221, Celgene Corporation) was the first IDH inhibitor approved by the Food and Drug Administration (FDA) for the treatment of relapsed or refractory (R/R) AML with *IDH2* mutation [201]. Phase I/II clinical trial (NCT01915498), conducted in 239 patients showed promising results with an overall response rate of 40.3% with 19.3% of patients who achieved a complete response [201]. Moreover, the drug exhibits a good toxicological profile., Like other targeted therapy they are characterized by few side effects that includes nausea, vomiting, diarrhoea, jaundice and decreased appetite [204]. Currently, ongoing clinical trials are investigating the use of enasidenib alone or in combination with standard chemotherapy drugs such as azacitidine in the treatment of hematologic malignancies as well as in glioma, cholangiocarcinoma, and chondrosarcoma (ClinicalTrials.gov NCT03683433, NCT02677922, NCT03839771) [205]. The other IDH inhibitor ivosidenib (Tibsovo^®^, AG-120, Agios Pharmaceuticals, Inc.) received FDA approval for the treatment of R/R AML carrying the IDH1 mutated form [200]. The result of a single-arm study (NCT02074839) provided valuable data regarding its efficacy and safety; indeed, 30.4% of patients achieved a complete or partial remission with a median response duration of 8.2 months [206]. In addition, 37% of AML patients became transfusion independent, and 21% had no residual detectable *IDH1* mutations [206]. The IDH1 inhibitor showed a favourable toxicity profile including fatigue, anaemia, nausea, diarrhoea, with a few relevant specific side effects such as alteration in heart rhythm and increase of serum aminotransferase levels [207]. Ivosidenib was also recently approved as first-line of treatment for newly diagnosed AML in patients over 75 years old [208] while preliminary results from phase III clinical trials seem to prove its clinical activity also in *IDH1* mutated cholangiocarcinoma [209]. Other small IDH inhibitors such as olutasidenib (FT-2102) are currently under investigation in phase I/II clinical trials for AML and myelodysplastic syndrome [202] as well as vorasidenib (AG-881), a pan inhibitor of both IDH1/2 enzymes, has been evaluated in glioma (NCT04164901) [199]. Despite the promising results of these IDH inhibitors, some studies reported clinical cases of acquired resistance, which was found associated with the occurrence of a new second-site *IDH2* mutations *in trans* or *in cis* at the level of the drug binding site [210], or with the emergence of an additional mutation that restored the R-2HG synthesis [211], while others exhibited the isoform switching phenomena from IDH1 to IDH2 and vice versa [212]. Moreover, the use of IDH inhibitors presents several challenges and their clinical application needs to be carefully evaluated. The R-2HG rise leads to concomitant NADPH consumption that may alter the ox-red cancer status making *IDH*-mutated cells more sensitive to standard chemo and radiotherapy due to the ROS burst [213,214,215,216,217]. In the same context, the DNA damage repair inhibition mediated by R-2HG makes *IDH* mutated tumours more vulnerable to alkylating drug treatment [218]. These specific biochemical features of *IDH* mutated cells can suggest that IDH inhibitors treatment may not be always advantageous and must be carefully evaluated on the basis of specific tumour metabolic characteristics [218,219,220,221]. 

An interesting new therapeutic strategy targeting *IDH* mutated cancers is represented by the development of IDH immune vaccines [222,223]. This approach is based on the evidence that glioma patients developed a Th-1 response against IDH1(R132H)^+^ tumours [222,223,224,225]. This inspired the development of a targeted- mutant IDH neoepitope vaccine to potentiate the response of the immune system against *IDH* mutated tumours. Two clinical trials are currently ongoing to test such immune strategy (NCT02193347, NCT02454634) [222,223]. The latter study has recently ended phase I, demonstrating the safety profile of peptide vaccine in grade III and IV astrocytomas patients [226]. 

Besides the inhibition of oncometabolite production, the other pharmacological approaches contrast the oncometabolites downstream effects. For instance, the glutaminase inhibitors were effective in reducing the growth of SDH and FH-deficient cancers [227,228,229] by lowering the glutamine needed to replenish TCA intermediates [227,229] including the R-2HG in *IDH* mutant tumours [230,231,232]. Conversely, high doses of α-KG may restore the hydroxylation activity of α-KGDDs enzymes by sweeping the oncometabolites in the binding site. However, only a few preclinical studies confirmed that the α-KG excess can effectively restore the PDHs as well as the TETs activity [136,233]. Another more established therapeutic option consisted of the use of demethylating agents to reverse the hypermethylated phenotype. In this context, the use of 5-azacitidine and its derivative decitabine were shown to reduce the methylation in *SDH* knock-out cells [136,234] as well as in *IDH* mutated gliomas [235,236,237,238]. This activity has been translated in clinical setting by reporting a better overall survival in *IDH* mutated AML patients treated with azacitidine/decitabine in combination with the BCL-2 inhibitor venetoclax [239,240]. The combination of azacitidine and different IDH inhibitors showed complete remission in AML patients suggesting a synergetic activity between the two drugs [200,201,202,241]. Following these promising results, other novel hypomethylating agents such as guadecitabine are currently under investigation in a phase II trial in SDH-deficient PCCs (ClinicalTrials.gov, NCT03165721).

## 5. Conclusions

Behind ATP production, billions of endosymbiotic evolutions led mitochondria to play a key role in the fine tuning of eukaryotic cell signalling and in determining the cellular fate. The discovery of the oncometabolites fumarate, succinate, and R-2HG, all belonging to the TCA cycle, have overwhelmingly reproposed the original Warburg theory according to which metabolic defect in mitochondria may be at the base of cancer development. The pivotal role of oncometabolites in driving malignant transformation through epigenetic modulation and pseudohypoxia effect goes beyond the genetics mechanisms underlying the overall importance of the metabolism studies for the comprehensive understanding of cancer cell development and progression. So far, the overall metabolic mechanisms of cancer disease remain to be fully understood, however, the elucidation of specific metabolic characteristics pointed out by oncometabolites inspired novel targeted therapeutic approaches that paved the way for controlling cancer growth by rewiring cancer cell metabolism.

## Figures and Tables

**Table 1:** Targeted therapies for *IDH* mutation.

Drug	Phase	Target	Mechanism of Action	Ref.
Enasidenib (AG-221) 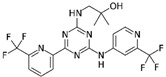	FDA approval	IDH2	Reversible, allosteric non-competitive inhibition via stabilization of the mutated IDH non-catalytic open conformation that prevents R-2HG formation [242].	[201]
Ivosidenib (AG-120) 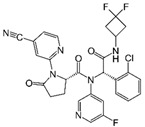	FDA approval	IDH1	Reversible, allosteric inhibition of IDH1 R132 mutants competing with the cofactor Mg ion and preventing the formation of the catalytically active protein conformation [243]	[200]
Olutasidenib (FT-2102) 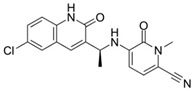	I/II	IDH1	Competitive inhibition at isocitrate-binding pocket blocking the conformational changes necessary for the catalysis.	[202]
BAY-1436032 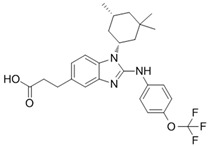	I	IDH1	Non-competitive, allosteric inhibition by binding at the interface of two monomers and stabilization of open inactive conformation.	[244,245,246]
Vorasidenib (AG-881) 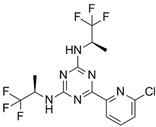	III	IDH1/2	Non-competitive, allosteric inhibition by binding at the interface of two monomers and stabilization of open inactive conformation.	[199]
IDH-305 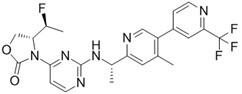	I	IDH1	Allosteric, non-competitive inhibition via stabilization of open, inactive enzyme dimer conformation (steric hindrance).	[198]
AGI-5198 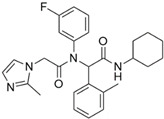	Pre-clinical	IDH1	Allosteric, competitive inhibition of α-KG.	[247,248]
AGI-6780 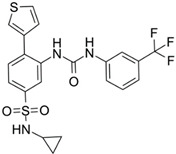	Pre-clinical	IDH2	Allosteric inhibition by binding at the monomers interface preventing the transition for the active enzyme conformation.	[249,250]
GSK321 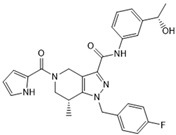	Pre-clinical	IDH1	Allosteric inhibition by blocking the enzyme in the inactive conformation.	[251]
PEPIDH1M vaccine	I	IDH1	T-helper-1 (T_H_1) responses are activated by presentation to major histocompatibility complexes (MHC) class II of the peptide encompassing the immunogenic epitope of the mutated IDH region.	[223]
IDH1 peptide vaccine	I	IDH1	IDH1(R132H)-specific peptide vaccine enhances T helper cell responses against tumours in synergism with the mutated IDH immunogenic epitope.	[222,226]
